# Cell cycle specific radiosensitisation by the disulfiram and copper complex

**DOI:** 10.18632/oncotarget.19539

**Published:** 2017-07-25

**Authors:** Mathias Tesson, Giorgio Anselmi, Caitlin Bell, Robert Mairs

**Affiliations:** ^1^ Radiation Oncology, Institute of Cancer Sciences, Wolfson Wohl Translational Cancer Research Center, University of Glasgow, Bearsden, Glasgow, UK; ^2^ Centre for Molecular and Cellular Biology of Inflammation, Peter Gorer Department of Immunobiology, Division of Immunology, Infection and Inflammatory Diseases, King’s College London, London, UK; ^3^ Cancer Research UK Beatson Institute, Bearsden, Glasgow, UK

**Keywords:** disulfiram, copper, radiosensitisation, DNA replication, gemcitabine

## Abstract

The disulfiram and copper complex (DSF:Cu) has emerged as a potent radiosensitising anti-cancer agent. The ability of copper to stabilise DSF in a planar conformation and to inhibit DNA replication enzymes stimulated our investigation of the effect of DSF:Cu on cell cycle regulation. Flow cytometry and immunoblotting were used to assess the effect of DSF:Cu on cell cycle progression of the neuroblastoma cell line SK-N-BE(2c) and the glioma cell line UVW. Treatment with 0.1 and 0.3 μM DSF:Cu inhibited DNA synthesis in SK-N-BE(2c) and UVW cells, respectively. The increased potency of ionising radiation treatment induced by DSF:Cu and/or gemcitabine was determined by clonogenic assay. Treatment with 0.3 μM DSF:Cu resulted in greater radiation kill, exemplified by dose enhancement factor values of 2.64 and 2.84 in SK-N-BE(2c) and UVW cells, respectively. Although DSF:Cu failed to sensitise S phase cells to irradiation, we observed that DSF:Cu radiosensitisation was potentiated by the S phase-specific cytotoxic drug gemcitabine. The efficacy of the combination treatment consisting of DSF:Cu, gemcitabine and ionising radiation was schedule-dependent. Together, these results describe cell cycle specific radiosensitisation by DSF:Cu. The well-established toxicity profiles of DSF and gemcitabine should facilitate their evaluation as a combination treatment in patients undergoing radiotherapy.

## INTRODUCTION

The dithiocarbamate disulfiram (DSF) has been used for several decades as a deterrent in the treatment of alcohol addiction [1]. Recently, the role of DSF in the treatment of cancer has been recognised although its mechanism of action has not been established. DSF has been shown to inhibit proteasome activity [2], angiogenesis [3], P-glycoprotein (P-gp) [4] and aldehyde dehydrogenase [5, 6]. Furthermore, DSF generates oxidative stress through the inhibition of NF-κB activation [7], by inhibition of superoxide dismutase (SOD) [8] and as a result of its induction of an increase in the ratio of oxidised glutathione to its reduced form [9]. It has also been shown that copper is required for DSF cytotoxicity [10] and that DSF acts as a copper ionophore [11, 12]. Therefore, intracellular copper deposition could be cytotoxic through the generation of oxidative stress resulting from Fenton reactions [producing cupric ions and hydroxyl radicals] or through enzymatic inhibition by virtue of copper’s binding to peptidic bonds. Moreover, copper complexes have been shown to intercalate between the base pairs of DNA owing to their planar conformation [13-15]. These reports raise the possibility that copper confers upon DSF a favourable conformation for DNA intercalation, resulting in alteration to the structure of DNA and cell death. It has been suggested that DSF and its reduced form, diethyldithiocarbamate, inhibit DNA synthesis through inhibition of DNA polymerase and ribonucleotide reductase [16, 17]. Finally, topoisomerase I, an enzyme whose activity relieves torsional stress generated at the replication fork during DNA replication, has been shown to be inhibited by DSF via binding to critical thiol residues [18]. A similar mechanism of inhibition of DNA synthesis has been reported for the nucleotide analogue gemcitabine which interacts with ribonucleotide reductase via cysteine [19].

Therefore, the cytotoxicity of the disulfiram and copper complex (DSF:Cu) may result from the generation of oxidative stress, inhibition of DNA replication or modulation of the activity of other critical cell regulatory pathways. Despite the multiplicity of potential targets, DSF-induced side-effects are reversible upon cessation of treatment and have been safely managed clinically since 1948 [20, 21].

DSF and its dithiocarbamate derivatives have been shown to be radioprotectors as measured by increased survival in yeast [22], decreased clonogenic cell kill [23], a longer survival in mice [24] and by a reduction in lipid peroxidation and DNA damage [25]. In contrast, dithiocarbamate derivatives have been reported to be radiosensitisers as measured by increased tumour growth delay in mice [26], increased clonogenic cell kill [23, 27-28] and by increased erythrocyte heamolysis [29]. However, these studies employed inappropriately high, hence clinically irrelevant, concentrations of DSF in experimental conditions uncontrolled for the presence of copper. Recently, we have shown that clinically pertinent concentrations of DSF:Cu sensitised tumour cells grown as monolayers, spheroids or xenografts to γ-radiation and radiopharmaceutical treatments [10]. The mechanism of radiosensitisation remains unknown. We hypothesised that DSF:Cu sensitised cancer cells to ionising radiation treatment via the modulation of cell cycle progression.

## RESULTS

### Proteasome-independent radiosensitisation by DSF:Cu

**Figure 1:**
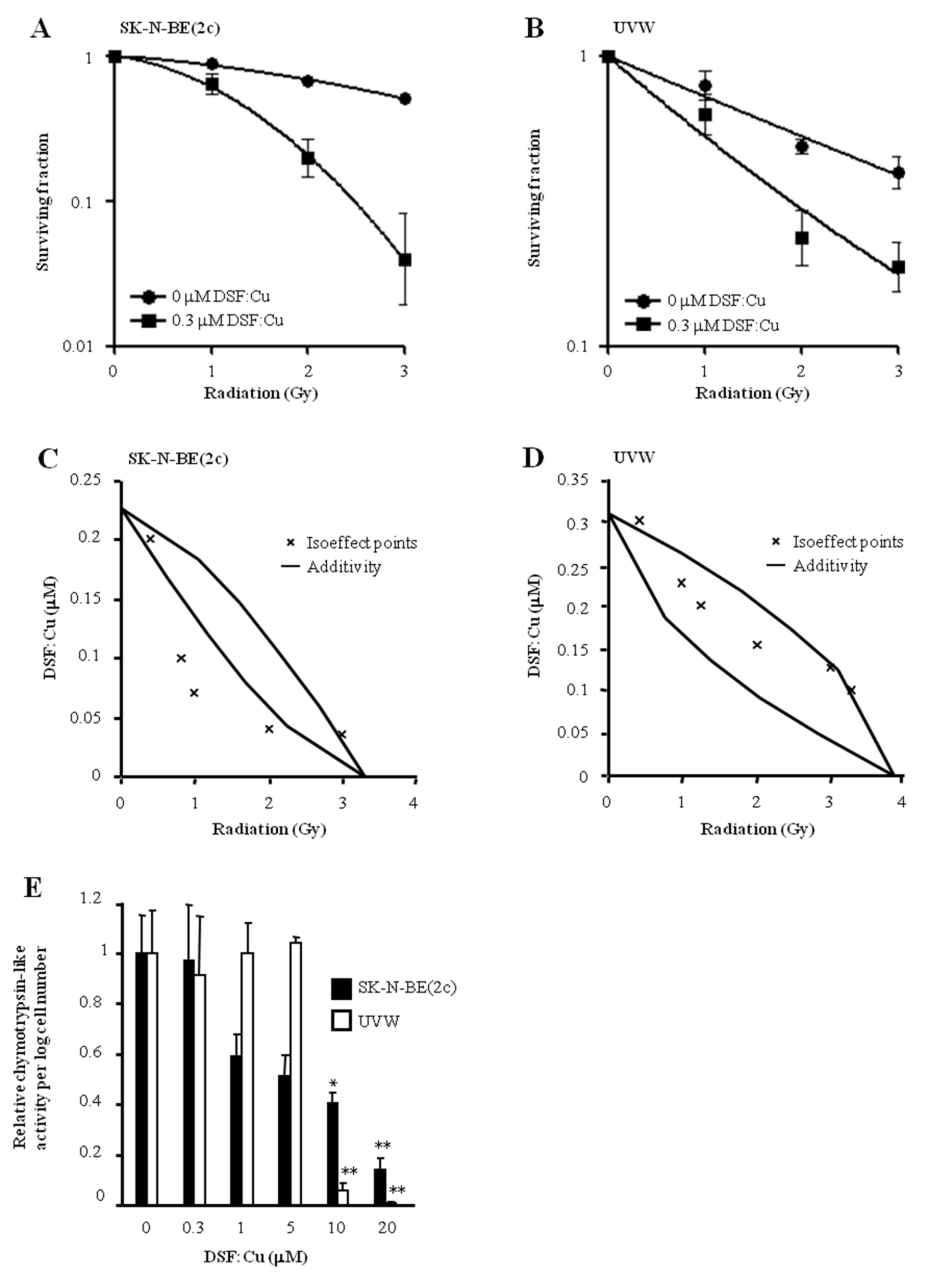
The effect of DSF:Cu on cellular radiosensitivity SK-N-BE(2c) **(A)** and UVW cells **(B)** were exposed to 0.3 μM DSF:Cu for 24 h and irradiated at the start of exposure to DSF:Cu. Data are means ± SEM, n=3. The nature of the interaction between IR and DSF:Cu was evaluated by isobologram analysis using SK-N-BE(2c) **(C)** and UVW cells **(D)**. **(E)** The effect of DSF:Cu on the chymotrypsin-like activity of the proteasome was evaluated. Data are means ± SEM, n=3. The independent t-test was used to compare the means of test groups with those of untreated control: ^*^P<0.05; ^**^P<0.01.

DSF:Cu enhanced the clonogenic cell kill induced by ionising radiation in SK-N-BE(2c) (Figure 1A) and UVW cells (Figure 1B). The IC_50_ values following irradiation decreased 2.64-fold or 2.84-fold in the presence of 0.3 μM DSF:Cu in SK-N-BE(2c) or UVW cells, respectively. The interaction between DSF:Cu and ionising radiation, as evaluated by isobologram analysis, was synergistic in SK-N-BE(2c) cells (Figure 1C) and additive in UVW cells (Figure 1D). Crucially, we previously reported that Cu alone does not influence radiosensitivity [10].

It has been shown that DSF:Cu inhibits proteasome activity [2, 30]. However, in SK-N-BE(2c) and UVW cells, proteasome activity was not inhibited following radiosensitising treatment consisting of 0.3 μM DSF:Cu (Figure 1E). This suggested that proteasome inhibition was not required for radiosensitisation by DSF:Cu. Together, these results confirmed the previously reported radiosensitising property of DSF:Cu [10], but indicated that a mechanism other than proteasome inhibition is responsible. The absence of a correlation between proteasome activity inhibition by DSF:Cu and cytotoxicity has also been suggested by others [31].

### DSF:Cu inhibits DNA synthesis

**Figure 2:**
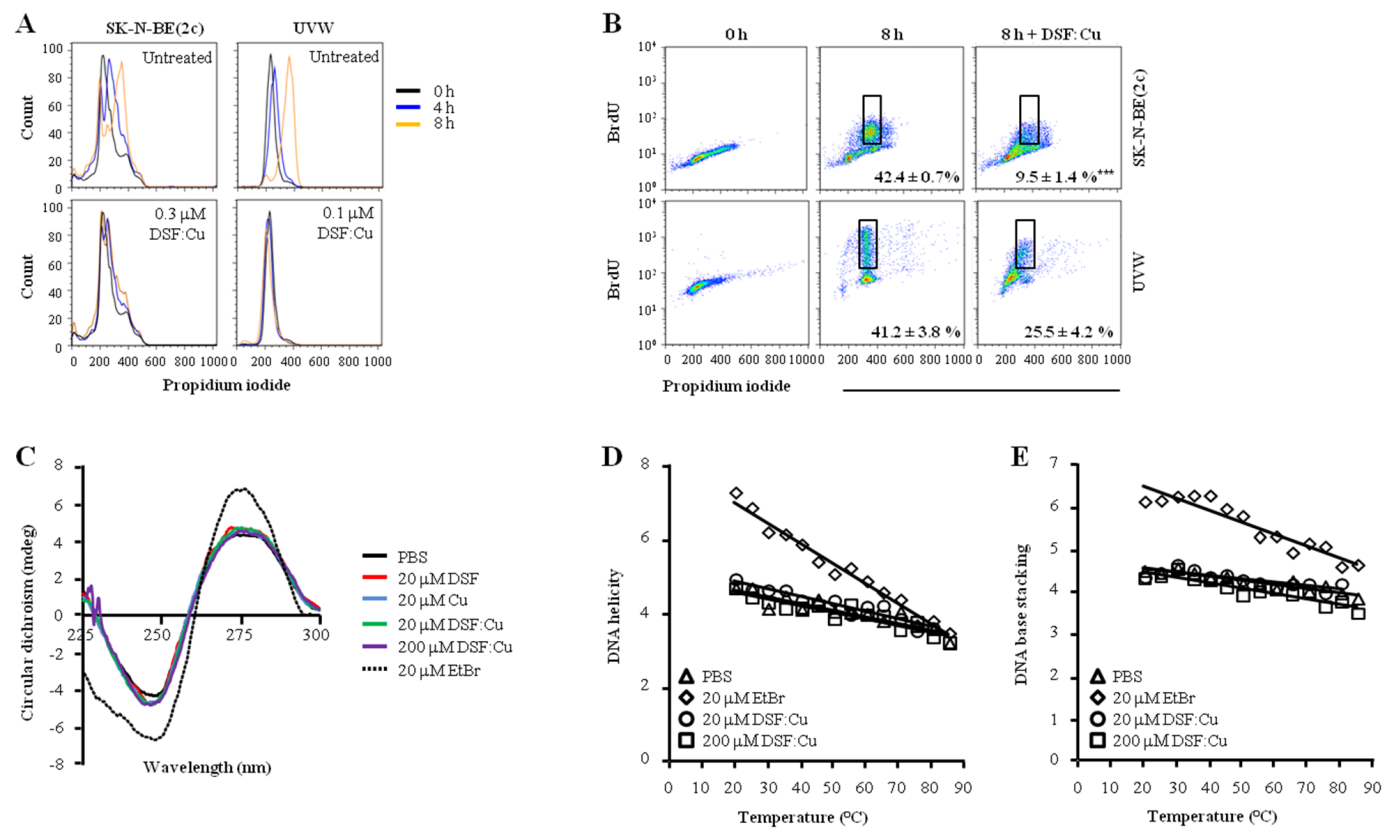
The effect of DSF:Cu on DNA synthesis Following release from thymidine block, synchronised SK-N-BE(2c) and UVW cells were exposed for 8 h to 0.3 or 0.1 μM DSF:Cu, respectively. The effect of DSF:Cu on DNA synthesis was monitored by flow cytometric analysis of propidium iodide-stained **(A)** or BrdU-stained **(B)** synchronised cells. The data are representatives of triplicate determinations. The proportions of BrdU-positive cells in G_2_ are means ± SEM, n=3. Independent t-test: ^***^ *P*<0.001. **(C)** The interaction between DSF:Cu and DNA was investigated by circular dichroism (CD) using treatment with ethidium bromide (EtBr) as a positive control. **(D, E)** The effect of DSF:Cu on the stability of the DNA molecule in response to heat treatment was investigated by CD. The absolute CD values at 245 nm (D, nadir of the negative wave) and at 275 nm (E, zenith of the positive wave) were plotted against temperature to measure change in helicity and base stacking, respectively.

Both SK-N-BE(2c) and UVW cells were arrested in early S phase by thymidine treatment. Their synchronous progression through S phase following release from thymidine block was indicated by the increase in propidium iodide intensity over 8 h (Figure 2A). DSF:Cu inhibited S phase progression (Figure 2A) and bromodeoxyuridine (BrdU) incorporation (Figure 2B). For instance, 8 h following release from the thymidine block, the proportion of G_2_ cells that incorporated BrdU was decreased 4.5-fold (*P*<0.001) in SK-N-BE(2c) cells and 1.6-fold in UVW cells by treatment with DSF:Cu (Figure 2B).

Copper complexes have been shown to be cytotoxic via binding to DNA [13-15]. Moreover, it has been suggested that DSF:Cu is a planar molecule [32], raising the possibility that DSF:Cu impedes DNA replication via insertion between the base pairs of DNA. Accordingly, we tested whether DSF:Cu could interact with DNA using circular dichroism (CD). In contrast to the known DNA intercalator ethidium bromide (EtBr), DSF:Cu did not modify the helicity nor the base stacking of calf thymus-DNA, as indicated by the lack of modification of the negative and positive waves of the CD spectrum, respectively (Figure 2C). Furthermore, the stability of calf thymus-DNA was tested by thermal denaturation in the presence or in the absence of DSF:Cu in order to determine whether interaction between DSF:Cu and calf thymus-DNA could occur via a mechanism other than intercalation. In control conditions without DSF:Cu, both helicity (Figure 2D) and base stacking (Figure 2E) of the DNA helix were lost as temperature increased. In contrast to the consequence of EtBr treatment, the rate of loss of DNA conformation due to increasing temperature was unaffected by DSF:Cu (Figure 2D and 2E). These results suggested that interaction between DSF:Cu and DNA is unlikely and that an alternative mechanism is responsible for the inhibition of DNA synthesis by DSF:Cu. One possibility is a negative regulatory impact of DSF:Cu upon enzymes responsible for DNA synthesis.

### DSF:Cu does not abrogate G_2_ arrest induced by ionising radiation

**Figure 3:**
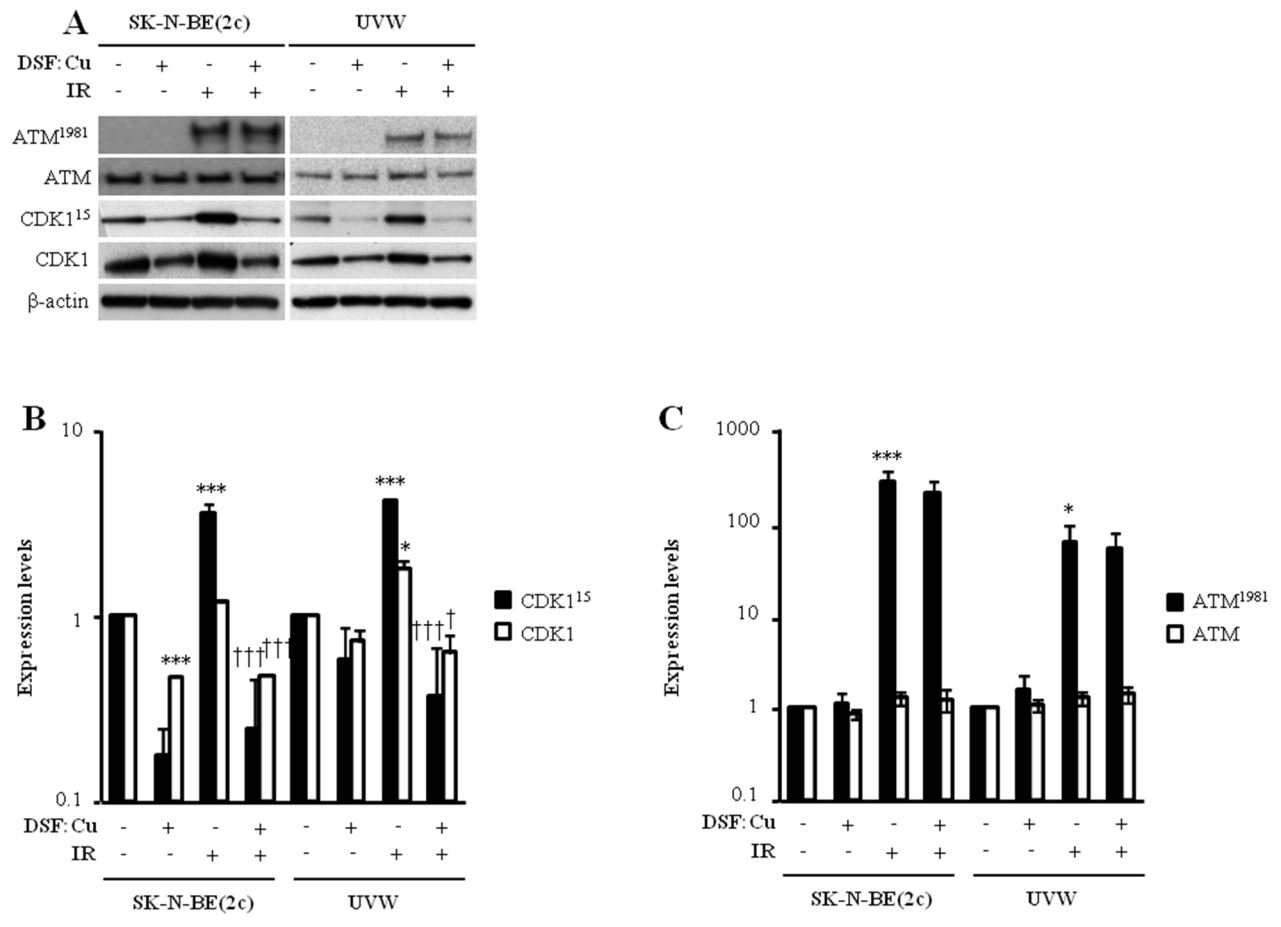
The effect of DSF:Cu on the phosphorylation of ATM and CDK1 induced by ionising radiation **(A)** The expression levels of ATM and ATM^1981^ were determined by immunoblotting 1 h following irradiation whereas CDK1 and CDK1^15^ expression levels were determined 12 h following irradiation. SK-N-BE(2c) and UVW cells were exposed to 0.3 μM DSF:Cu and irradiated with 5 Gy at the start of exposure to DSF:Cu. The immunoblots are representative examples of triplicate determinations. **(B, C)** Histogram data are fold changes in expression levels above background compared with untreated controls and normalised to the loading control β-actin. Data are means ± SEM, n=3. One-way ANOVA with Bonferroni correction was used to compare the means of the test groups with those of the control (*) or with those of the irradiated group (†). One symbol *P*<0.05, two symbols *P*<0.01; three symbols *P*<0.001.

We next evaluated whether DSF:Cu affected cell cycle progression through the G_2_ phase of the cell cycle. Exposure to DSF:Cu for 12 h resulted in increased proportions of SK-N-BE(2c) and UVW cells in G_2_-M from 30.5% to 34.5% and from 23.0% to 33.9% (*P*<0.01), respectively (Table 1), indicating G_2_ arrest. This conclusion was supported by the observed decrease in the level of expression of total CDK1 following treatment of SK-N-BE(2c) (*P*<0.001) or UVW cells with DSF:Cu (Figure 3A, 3B). Decreased CDK1 expression has previously been shown to prevent entry into mitosis [33, 34].

**Table 1:** The effect of DSF:Cu and ionising radiation on cellular accumulation at the G_2_ checkpoint.

	Control	DSF:Cu	5 Gy	DSF:Cu + 5 Gy
**SK-N-BE(2c)**	30.5 ± 0.63	34.5 ± 1.63	53.7 ± 5.05^*^	40.4 ± 0.51^¶^
**UVW**	23.0 ± 0.83	33.9 ± 2.57^**^	56.1 ± 1.40^***^	44.0 ± 0.50^††¶¶^

The percentages of unsynchronised SK-N-BE(2c) and UVW cells in G2-M, 12 h after irradiation and exposure to 0.3 μM DSF:Cu, were determined by FACS analysis of propidium-iodide-stained cells. Data are means ± SEM, n=3. One-way ANOVA with Bonferroni correction was used to compare the means of the test groups with those of the untreated control (*), with those of the irradiated group (†) or compared with those treated with DSF:Cu alone (¶). One symbol: *P*<0.05; two symbols: *P*<0.01; three symbols: *P*<0.001.

One of the hallmarks of the cellular response to ionising radiation is the activation of the G_2_ checkpoint prior to entry into mitosis. To determine whether radiosensitisation by DSF:Cu was due to the inhibition of G_2_ checkpoint activation following irradiation, the distribution of unsynchronised SK-N-BE(2c) and UVW cells throughout the phases of the cell cycle was assessed 12 h after irradiation in the absence or in the presence of DSF:Cu. Irradiation alone resulted in the activation of the G_2_ checkpoint in unsynchronised SK-N-BE(2c) and UVW cells (Table 1), as indicated by increases in the G_2_-M populations from 30.5% to 53.7% (*P*<0.05) and from 23.0% to 56.1% (*P*<0.001), respectively. In comparison with irradiation alone, the G_2_-M populations, following irradiation in the presence of 0.3 μM DSF:Cu, were decreased from 53.7% to 40.4% in SK-N-BE(2c) cells and from 56.1% to 44.0% (*P*<0.01) in UVW cells (Table 1), indicating that DSF:Cu may inhibit irradiation-induced G_2_ arrest. The activation of G_2_ arrest was also evaluated by immunoblotting of ATM^1981^ and CDK1^15^ (Figure 3). ATM^1981^ detects DNA damage in the form of double-strand breaks (DSB) and activates the G_2_ checkpoint as well as DSB repair [35]. CDK1^15^, a downstream effector of ATM^1981^, prevents entry into mitosis to allow for the repair of DNA damage [35]. The G_2_ checkpoint was activated following irradiation, as indicated by an increase in expression level of ATM^1981^ (*P*<0.05) and CDK1^15^ (*P*<0.001) in SK-N-BE(2c) and UVW cells (Figure 3B, 3C). In comparison with irradiation alone, expression of ATM^1981^ was unaltered by irradiation in the presence of DSF:Cu (Figure 3C), suggesting that DSF:Cu does not affect DSB detection following irradiation. In contrast, expression of CDK1^15^ was reduced (*P*<0.001) following irradiation in the presence of DSF:Cu in comparison with irradiation alone (Figure 3B). However, the decrease in CDK1^15^ expression is likely due to a decrease in total CDK1 expression.

We reasoned that if DSF:Cu suppressed the phosphorylation of CDK1 at tyrosine 15 as well as reducing the expression level of total CDK1, the levels of total CDK1 may be too low to trigger the mitotic process. Consequently, we anticipated that the apparent inhibition of the irradiation-induced G_2_ arrest may not be due to a bypass of G_2_ arrest but, instead, to the failure to complete S phase before entry into G_2_. Therefore, we studied cell cycle progression using synchronised populations of SK-N-BE(2c) and UVW cells. Synchronised cells were irradiated at the S-G_2_ border and immediately exposed to DSF:Cu. Cell cycle profiles were obtained 6 h after the commencement of irradiation and DSF:Cu treatments. At this timepoint, 30.5% of untreated SK-N-BE(2c) cells and 86.5% of untreated UVW cells had reached G_1_, indicating progress through G_2_ and mitosis (Table 2). In comparison, there were fewer SK-N-BE(2c) (17.2%, *P*<0.001) and UVW (4.6%, *P*<0.001) cells in G_1_ following DSF:Cu treatment alone (Table 2). The decrease in the proportion of cells in G_1_ following DSF:Cu treatment was accompanied by an increase in the proportion of SK-N-BE(2c) and UVW cells in G_2_, indicating failure to progress through mitosis (Table 2). These results corroborated the observation made in Table 1 that DSF:Cu causes arrest in G_2_.

**Table 2:** The effect of DSF:Cu and ionising radiation on the progression of synchronised cells through G_2_.

	Control	DSF:Cu	5 Gy	DSF:Cu + 5 Gy
**SK-N-BE(2c)**				
G1	30.5 ± 0.70	17.2 ± 0.27^***^	16.0 ± 0.79^***^	17.2 ± 0.12^***^
S	18.8 ± 0.31	28.4 ± 0.48^***^	17.0 ± 1.10	28.3 ± 0.76^***^
G2	33.7 ± 0.40	39.2 ± 0.62	42.4 ± 3.15^*^	37.3 ± 1.09
**UVW**				
G1	86.5 ± 0.18	4.6 ± 0.70^***^	13.2 ± 1.49^***^	5.1 ± 0.12^***^
S	2.4 ± 0.08	1.2 ± 0.06	1.6 ± 0.06	1.8 ± 0.53
G2	9.6 ± 0.33	93.8 ± 0.79^***^	84.3 ± 1.62^***^	92.8 ± 1.65^***^

The percentages of synchronised SK-N-BE(2c) and UVW cells in G_1_, S and G_2_ were determined by FACS analysis of propidium-iodide-stained cells 6 h after irradiation at the S-G_2_ border and treatment with 0.3 μM DSF:Cu. Data are means ± SEM, n=3. One-way ANOVA with Bonferroni correction was used to compare the means of the test groups with those of the untreated control (*). One symbol: *P*<0.05; two symbols: *P*<0.01; three symbols: *P*<0.001.

Irradiation at the S-G_2_ border increased the proportion of SK-N-BE(2c) (*P*<0.05) and UVW (*P*<0.001) cells in G_2_ (Table 2). The increase in the proportion of cells in G_2_ following irradiation was accompanied by a decrease in the proportion of both SK-N-BE(2c) (*P*<0.001) and UVW (*P*<0.001) cells in G_1_. There was no inhibition of irradiation-induced cellular accumulation in G_2_ by DSF:Cu, indicated by similar proportions of cells in G_2_ and G_1_ in the irradiated group and in the group irradiated in the presence of DSF:Cu (Table 2). These observations indicated the failure of synchronised cells to bypass the irradiation-induced G_2_ arrest in the presence of DSF:Cu.

Taken together, these results indicated the ability of DSF:Cu treatment to cause accumulation of cells in G_2_ and to inhibit DNA synthesis. The apparent prevention of the irradiation-induced accumulation of unsynchronised cells in G_2_ by DSF:Cu (Table 1) is likely due to a cytostatic effect of DSF:Cu on cell cycle progression through S phase.

### Enhancement of cellular radiosensitivity by combination of DSF:Cu with the nucleotide analogue gemcitabine

**Figure 4:**
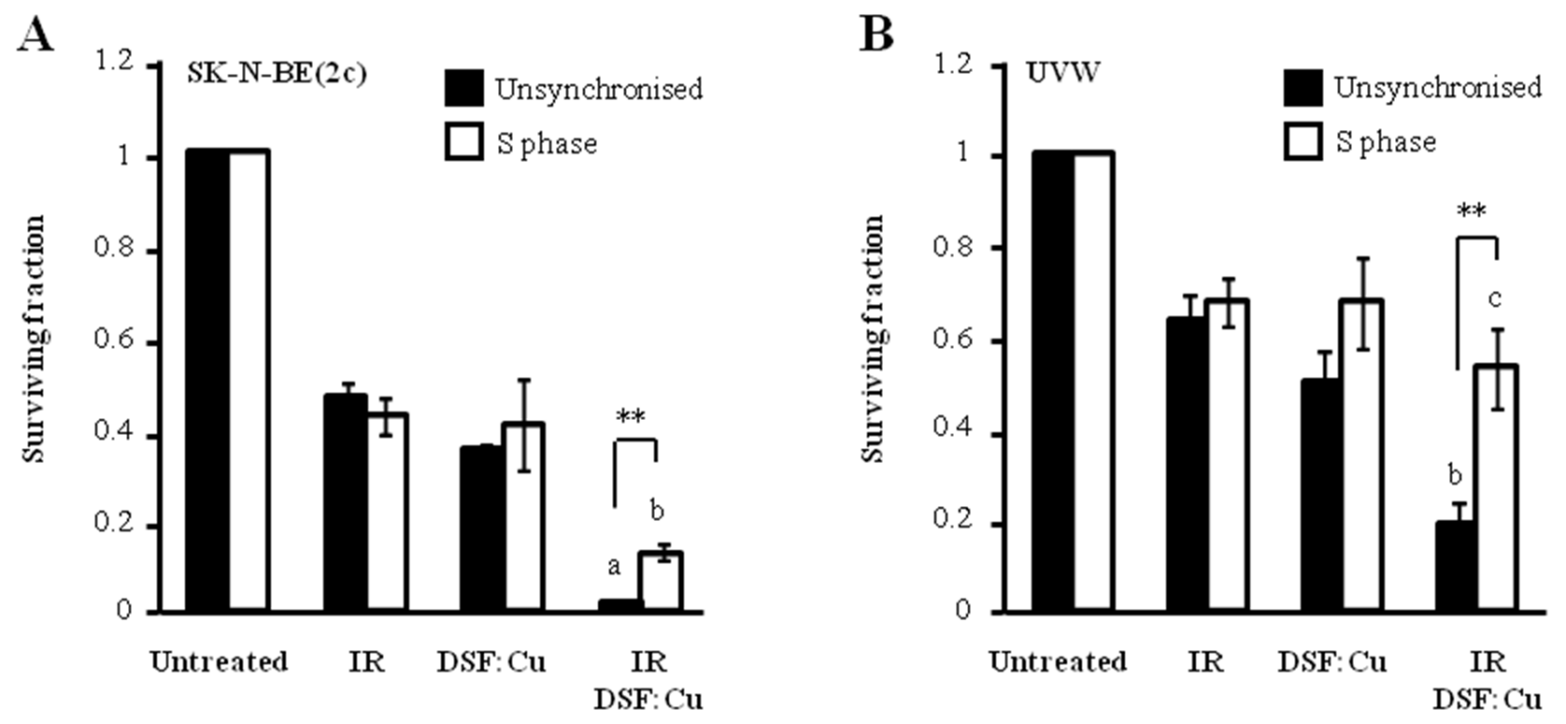
The effect of DSF:Cu on cellular radiosensitivity in S phase **(A)** Unsynchronised SK-N-BE(2c) cells were irradiated with 3 Gy in the absence or the presence of 0.15 μM DSF:Cu. Alternatively, following synchronisation, SK-N-BE(2c) cells were irradiated at the start of S phase with 2 Gy in the absence or the presence of 0.15 μM DSF:Cu. Data are means ± SEM, n=3. **(B)** Unsynchronised UVW cells were irradiated with 3 Gy in the absence or the presence of 0.1 μM DSF:Cu. Alternatively, following synchronisation, UVW cells were irradiated at the start of S phase with 1 Gy in the absence or the presence of 0.1 μM DSF:Cu. Data are means ± SEM, n=3. One-way ANOVA with Bonferroni correction was used to compare the means of the combination treatment with those treated with either single agent (a, *P*<0.001; b, *P*<0.01, c, non-significant). The independent t-test was used to compare the means of the combination treatment in unsynchronised cells versus those of S phase cells (^**^P<0.01).

Our results indicate that DSF:Cu is a radiosensitiser and that it inhibits DNA replication. However, the radiosensitivity of cells in S phase relies on a moving replication fork for the creation of lethal DSB in DNA [36]. Therefore, the radiosensitivity of S phase cells was evaluated in the presence of DSF:Cu. A clonogenic cell kill of 55% was achieved by irradiating unsynchronised SK-N-BE(2c) cells with 3 Gy whereas 2 Gy was sufficient to achieve the same kill in SK-N-BE(2c) cells synchronised in early S phase (Figure 4A). Similarly, 40% kill of UVW clonogens was achieved by irradiating unsynchronised UVW cells with 3 Gy whereas 1 Gy was sufficient to achieve the same kill of UVW cells synchronised in early S phase (Figure 4B). These observations indicated that cells in early S phase are more sensitive to irradiation than unsynchronised populations. DSF:Cu radiosensitised SK-N-BE(2c) cells in S phase to a lesser degree than unsynchronised cells (*P*<0.01) whereas DSF:Cu failed to radiosensitise UVW cells in S phase (*P*<0.001) (Figure 4). These data indicated the resistance of cells in S phase to combination treatment consisting of ionising radiation and DSF:Cu.

Gemcitabine is a nucleotide analogue which kills cancer cells specifically in S phase. DSF:Cu has been shown to enhance gemcitabine efficacy through the inhibition of NF-κB [37], the generation of oxidative stress [38] or the eradication of aldehyde dehydrogenase-positive cancer cells [5, 6, 39]. Since S phase cells showed resistance to treatment consisting of ionising radiation and DSF:Cu, we hypothesised that gemcitabine treatment could increase its efficacy by targeting S phase cells. In view of the fact that gemcitabine toxicity to S phase cells depends on its incorporation into DNA and that DSF:Cu inhibits DNA synthesis, we hypothesised that modulation by gemcitabine of the efficacy of the treatment consisting of ionising radiation and DSF:Cu may be schedule-dependent. Preliminary investigations indicated that Cu does not influence the sensitivity of SK-N-BE(2c) nor SH-SY5Y cells to gemcitabine treatment (Supplementary Figure 1).

**Figure 5:**
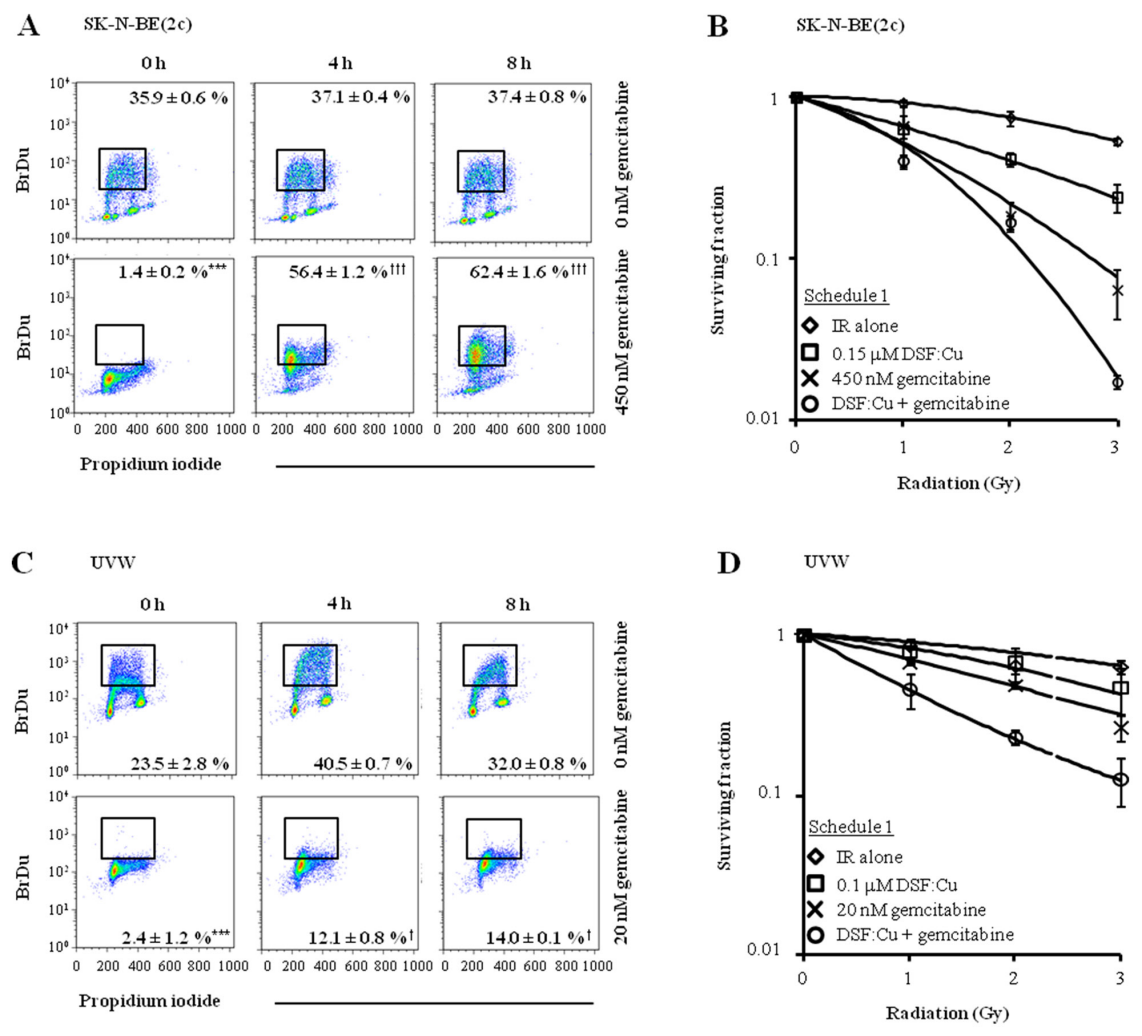
The effect of drug administration according to schedule 1 on clonogenic survival of SK-N-BE(2c) and UVW cells **(A, C)** Unsynchronised SK-N-BE(2c) and UVW cells were exposed, for 24 h, to 450 nM or 20 nM gemcitabine, respectively. These concentrations of gemcitabine corresponded to their IC_50_ values. The ability of SK-N-BE(2c) and UVW cells to incorporate BrdU into the DNA was measured by flow cytometry 0, 4 and 8 h following removal of gemcitabine from the culture medium. The data are representative examples of triplicate determinations. The proportions of BrdU-positive cells are means ± SEM, n=3. One-way ANOVA with Bonferroni correction was used to compare the means of the test groups with those of the control (*) or with those of group treated with gemcitabine at the 0 h time-point (†). One symbol *P*<0.05, 3 symbols *P*<0.001. **(B, D)** Schedule 1 consisted of pre-treatment of cells with gemcitabine for 24 h immediately followed by treatment with DSF:Cu for 8 h. The cells were irradiated at the commencement of exposure to DSF:Cu. Data are means ± SEM, n=3.

Exposure of SK-N-BE(2c) or UVW cells to gemcitabine for 24 h inhibited DNA synthesis, indicated by a significant decrease in the proportion of cells incorporating BrdU (*P*<0.001) (Figure 5A and 5C). However, in SK-N-BE(2c) cells, BrdU positivity significantly increased from 1.4 ± 0.2 to 62.4 ± 1.6 % (*P*<0.001) 8 h after removal of gemcitabine from the medium, indicating the reversible nature of the inhibition of DNA synthesis in SK-N-BE(2c) cells by gemcitabine (Figure 5A). In UVW cells, although BrdU positivity significantly increased from 2.4 ± 1.2 to 14.0 ± 0.1 % (*P*<0.05) 8 h following removal of gemcitabine from the medium, there was no increase in BrdU-positive cells from 4 to 8 h, indicating that DNA synthesis did not restart within 8 h of removal of gemcitabine (Figure 5C). Pre-treatment of unsynchronised SK-N-BE(2c) and UVW cells with gemcitabine for 24 h before exposure to DSF:Cu for 8 h (schedule 1) resulted in increased radiation DEF values in comparison with treatment with gemcitabine or DSF:Cu alone (Figure 5B, 5D, Table 3). The nature of the interaction between DSF:Cu, gemcitabine and irradiation was evaluated by combination index (CI) analysis. Synergism was indicated by CI values less than 1 at toxicity levels greater than 0.5 in SK-N-BE(2c) cells and greater than 0.25 in UVW cells (Table 4). These results indicated that re-initiation of DNA synthesis following removal of gemcitabine from the culture medium in SK-N-BE(2c) cells, but not in UVW cells, did not affect the efficacy of drug administration according to schedule 1.

**Table 3:** Radiation dose enhancement values at the 90% toxicity level.

	DSF:Cu	Gemcitabine	DSF:Cu + gemcitabine
**SK-N-BE(2c)**			
Schedule 1	1.59 ± 0.34	2.48 ± 0.28	2.97 ± 0.43
Schedule 2	1.64 ± 0.45	1.42 ± 0.09	2.59 ± 0.49
Schedule 3	2.39 ± 0.49	2.29 ± 0.19	4.50 ± 0.83
**UVW**			
Schedule 1	1.45 ± 0.35	2.17 ± 0.24	3.35 ± 0.35
Schedule 2	1.32 ± 0.26	1.41 ± 0.09	2.06 ± 0.45
Schedule 3	0.88 ± 0.19	1.15 ± 0.49	0.94 ± 0.05

The linear-quadratic equation was used to calculate the radiation dose enhancement factor at the 90% toxicity level following treatment with DSF:Cu, gemcitabine and ionising radiation according to administration schedules 1, 2 and 3. For SK-N-BE(2c) cells, the DSF:Cu and gemcitabine concentrations were 0.15 μM and 450 nM, respectively. For UVW cells, the DSF:Cu and gemcitabine concentrations were 0.1 μM and 20 nM, respectively. Data are means ± SEM, n=3.

**Table 4:** Combination indices describing the nature of the interaction between gemcitabine, DSF:Cu and ionising radiation

SK-N-BE(2c)			
Affected fraction	Schedule 1	Schedule 2	Schedule 3
0.25	2.67	2.49	2.54
0.5	1.37	1.33	1.31
0.75	0.82	0.81	0.81
0.95	0.42	0.42	0.46

**UVW**			
**Affected fraction**	**Schedule 1**	**Schedule 2**	**Schedule 3**
0.25	0.96	1.81	2.25
0.5	0.82	1.10	3.36
0.75	0.73	0.79	5.21
0.95	0.65	0.62	11.7

**SH-SY5Y**			
**Affected fraction**	**Schedule 1**	**Schedule 2**	**Schedule 3**
0.25	2.87	4.65	3.08
0.5	1.55	2.91	2.63
0.75	1.11	2.17	2.86
0.95	0.91	1.96	4.71

**T98G**			
**Affected fraction**	**Schedule 1**	**Schedule 2**	**Schedule 3**
0.25	3.55	5.53	3.21
0.5	1.69	2.13	2.80
0.75	1.13	1.36	3.96
0.95	0.92	1.04	11.3

The clonogenic assay was used to construct radiation survival curves. SK-N-BE(2c): ionising radiation, 1-2-3 Gy, 0.15 μM DSF:Cu, 450 nM gemcitabine; UVW: ionising radiation, 1-2-3 Gy, 0.1 μM DSF:Cu, 20 nM gemcitabine; SH-SY5Y: ionising radiation, 0.5-1-1.5 Gy, 0.05 μM DSF:Cu, 40 nM gemcitabine; T98G: ionising radiation, 1-2-3 Gy, 1 μM DSF:Cu, 50 nM gemcitabine. Combination index analysis was carried out to evaluate the nature of the interaction between ionising radiation, DSF:Cu and gemcitabine following administration schedules 1, 2 and 3.

**Figure 6:**
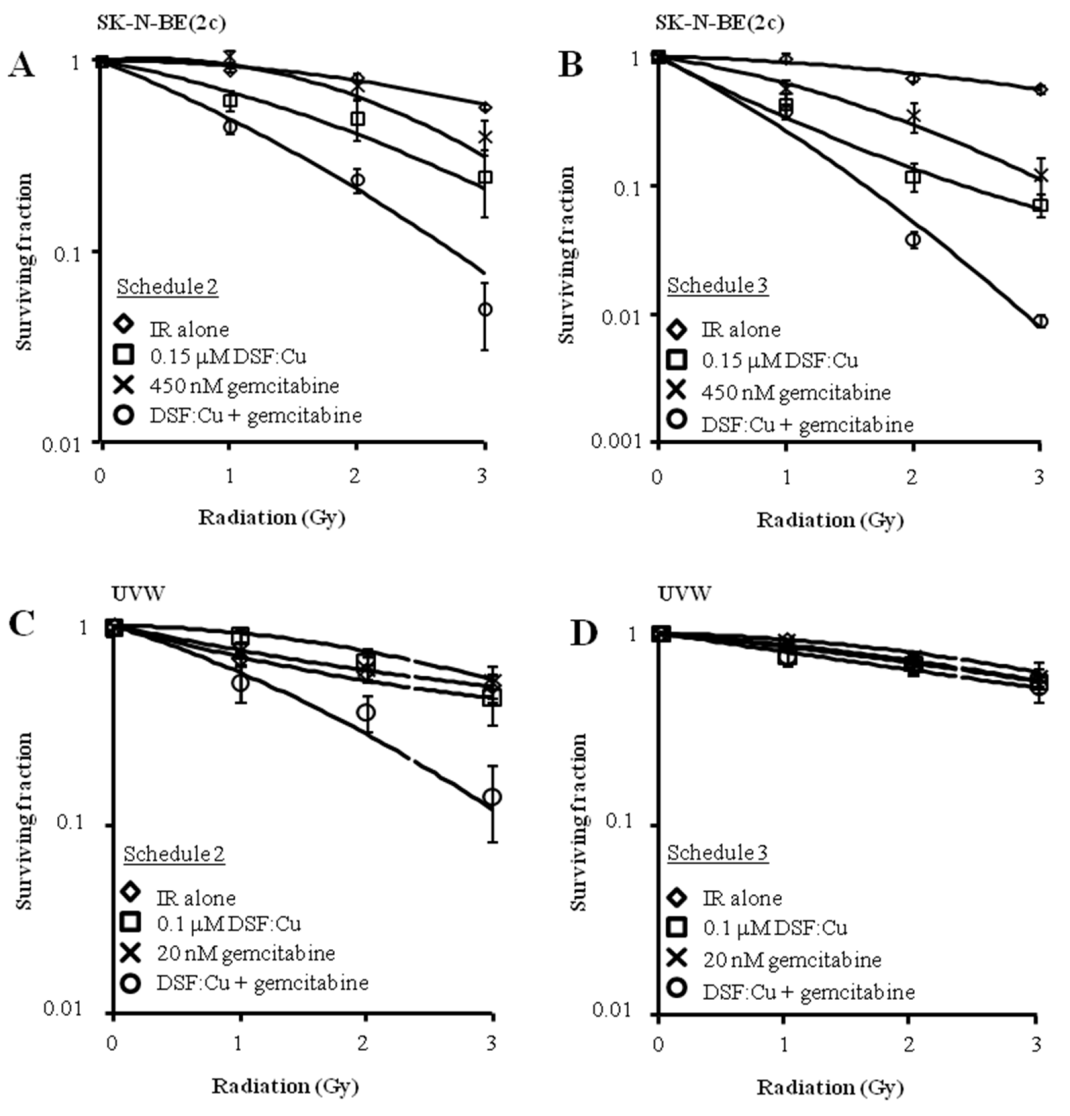
The effect of drug administration according to schedules 2 and 3 on clonogenic survival of SK-N-BE(2c) and UVW cells **(A, C)** Schedule 2 consisted of pre-treatment of cells with DSF:Cu for 8 h, with irradiation at the commencement of exposure to DSF:Cu, immediately followed by treatment with gemcitabine for 24 h. Data are means ± SEM, n=3. **(B, D)** Schedule 3 consisted of simultaneous treatment of cells with gemcitabine for 24 h and DSF:Cu for 8 h, with irradiation at the commencement of drug treatment. Data are means ± SEM, n=3.

Next, the efficacy of alternative schedules of drug administration was evaluated. Pre-treatment of unsynchronised SK-N-BE(2c) and UVW cells for 8 h with DSF:Cu followed by exposure to gemcitabine for 24 h (schedule 2) resulted in increased radiation DEF values in comparison with treatment with gemcitabine or DSF:Cu alone (Figure 6A, 6C, Table 3). Synergism was indicated by CI values less than 1 at toxicity levels greater than 0.5 in SK-N-BE(2c) and UVW cells (Table 4). The simultaneous treatment with gemcitabine, DSF:Cu and ionising radiation (schedule 3) resulted in increased radiation DEF values in comparison with treatment with gemcitabine or DSF:Cu alone in SK-N-BE(2c) cells (Figure 6B, Table 3), but not in UVW cells (Figure 6D, Table 3). Accordingly, synergism was observed only in SK-N-BE(2c) cells as indicated by CI values less than 1 at toxicity levels greater than 0.5 (Table 4).

The nature of the interaction between ionising radiation, DSF:Cu and gemcitabine was further evaluated in the neuroblastoma cell line SH-SY5Y and the glioma cell line T98G. In agreement with the observations made using UVW cells, the simultaneous treatment with gemcitabine, DSF:Cu and ionising radiation (schedule 3) resulted in an antagonistic interaction in SH-SY5Y and T98G, as indicated by CI values greater than 1 (Table 4). For both alternate schedules 2 and 3, there was a trend towards an additive interaction between gemcitabine, DSF:Cu and ionising radiation in SH-SY5Y and T98G at toxicity levels greater than 0.75 (Table 4). However, pre-treatment of SH-SY5Y with DSF:Cu and ionising radiation prior gemcitabine treatment (schedule 2) did not result in an additive interaction despite CI values decreasing towards additivity with increasing toxicity levels (Table 4).

**Figure 7:**
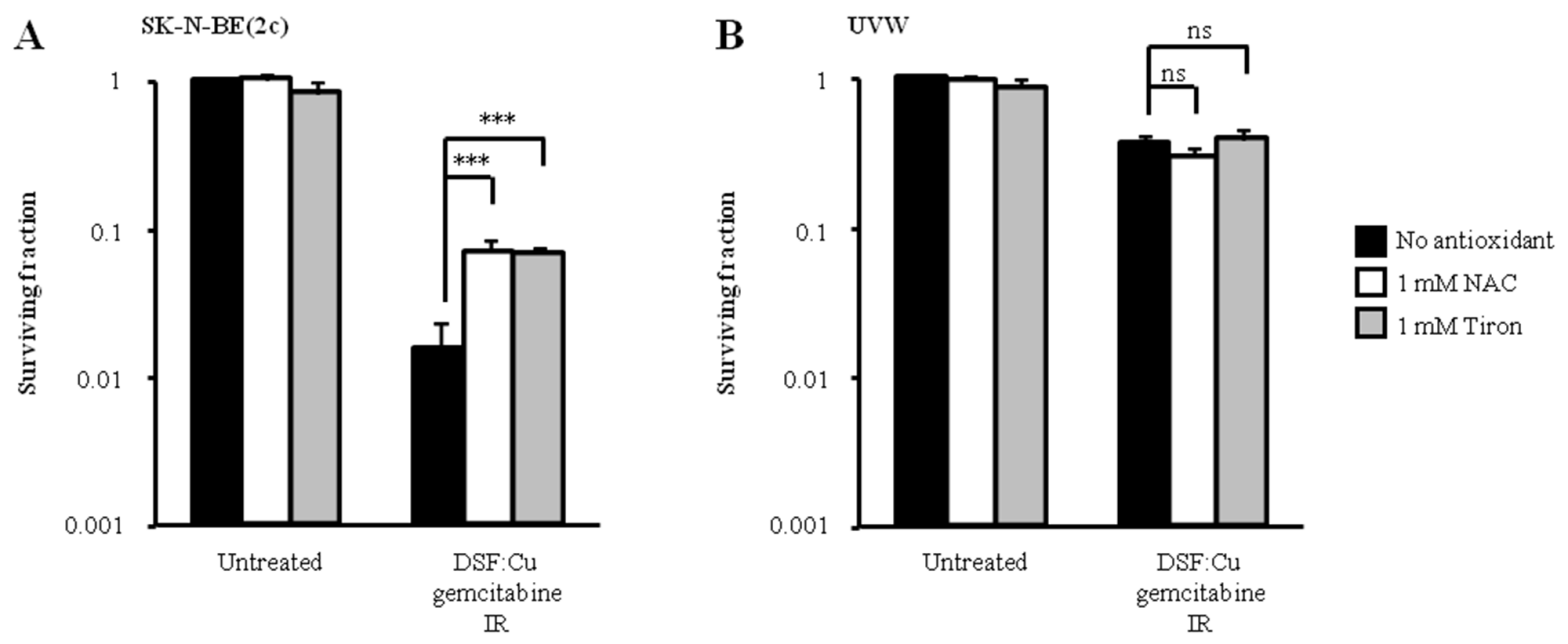
The effect of antioxidants on clonogenic survival following treatment administration schedule 3 **(A)** SK-N-BE(2c) cells were irradiated with 3 Gy and simultaneously treated with 0.15 μM DSF:Cu and 450 nM gemcitabine in the presence of 1 mM N-acetyl-cysteine (NAC) or 1 mM Tiron. **(B)** UVW cells were irradiated with 3 Gy and simultaneously treated with 0.1 μM DSF:Cu and 20 nM gemcitabine in the presence of 1 mM NAC or 1 mM Tiron. Data are means ± SEM, n=3. The independent t-test was used to compare the means of the groups treated in the absence of NAC or Tiron versus those treated in the presence of NAC or Tiron. ***P<0.001; ns, non-significant.

Since others have shown that the enhancement of gemcitabine cytotoxicity by DSF:Cu may be due to the generation of oxidative stress, we hypothesised that the difference in sensitivity to schedule 3 of drug administration between SK-N-BE(2c) and UVW cells may be due to their relative sensitivity to oxidative stress. It has also been shown that DSF induces the formation of reactive oxygen species (ROS) [5, 40]. In these studies, the generation of ROS following exposure of cells to DSF:Cu was prevented by treatment with the ROS scavenger N-acetyl cysteine (NAC) [5, 40]. Therefore we tested the sensitivity of SK-N-BE(2c) and UVW cells to schedule 3 in the absence and in the presence of the ROS scavenger NAC. However, since DSF interacts with thiols, the putative increased survival resulting from the presence of NAC may be due to direct interaction between NAC and DSF:Cu and not to the scavenging of ROS. Therefore, we also tested our hypothesis using the antioxidant Tiron whose mechanism of action is independent of thiol interaction [41]. We observed that the toxicity to SK-N-BE(2c) cells of schedule 3 treatment was reduced 10-fold (*P*<0.01) by treatment with 1 mM NAC or Tiron (Figure 7A). In contrast, NAC or Tiron failed to enhance the survival of UVW cells subjected to combination treatment using schedule 3 (Figure 7B). The enhanced kill of SK-N-BE(2c) cells, but not of UVW cells, induced by schedule 3 treatment may be due to a greater oxidative stress generated in SK-N-BE(2c) cells. In turn, the heightened sensitivity of SK-N-BE(2c) cells to oxidative stress may explain the synergistic interaction observed between ionising radiation and DSF:Cu in comparison with the additive interaction observed in UVW cells (Figure 1B, 1D). Alternatively, UVW cells may express higher levels of antioxidants or repair DNA more efficiently.

## DISCUSSION

**Figure 8:**
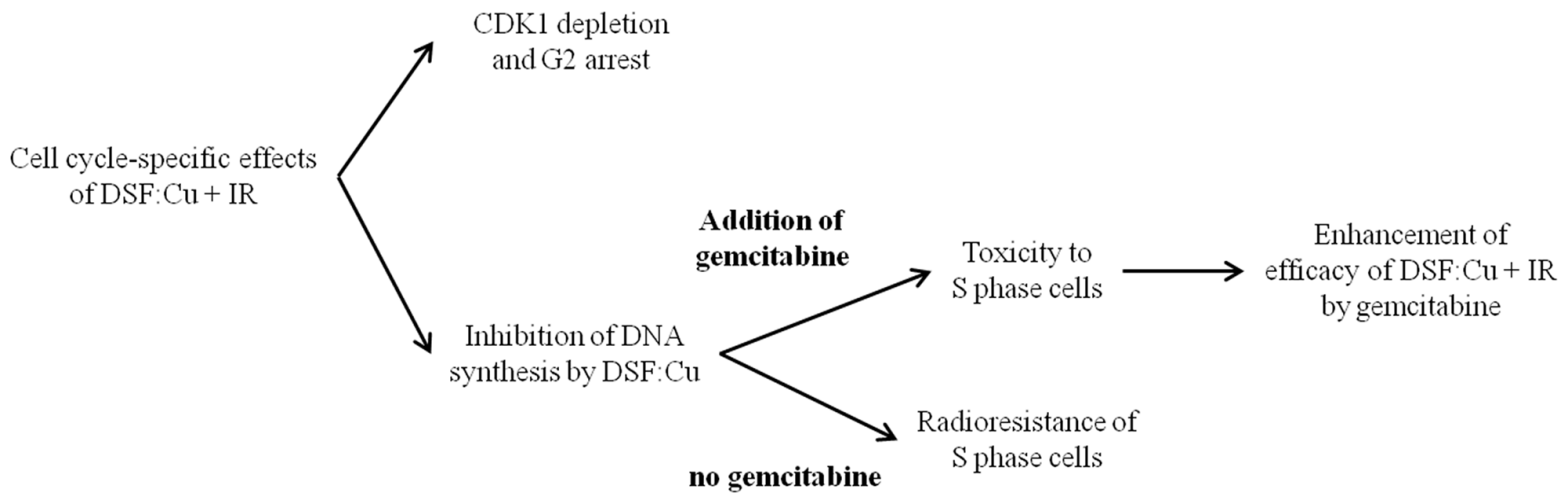
The summary of the strategy employed to enhance radiosensitisation by DSF:Cu

In summary, we report that DSF:Cu treatment sensitised cancer cells to ionising radiation via a mechanism independent of proteasome activity. The effects of DSF:Cu on cell cycle progression were manifest by inhibition of DNA synthesis, CDK1 depletion and blockade of entry into mitosis. Finally, the failure to radiosensitise cells in S phase by DSF:Cu was overcome by gemcitabine treatment in a cell-dependent and schedule-dependent manner. The cell cycle-specific effects of DSF:Cu and the strategy to enhance radiosensitisation by DSF:Cu are summarised in Figure 8.

Following exposure to stress, such as radiation, NF-κB is activated by phopsphorylation-induced proteasomal degradation of its inhibitor IkB [42]. NF-κB activation has been associated with radioresistance [43], notably via induction of anti-apoptotic and antioxidant gene expression [44, 45]. Furthermore, DSF has been shown to inhibit the proteasome [2] and NF-κB activation [7]. We have previously shown that specific proteasome inhibition, using bortezomib, is a potent radiosensitising strategy [46, 47]. However, in the present study, radiosensitisation by DSF:Cu could not be assigned to proteasome inhibition. Since NF-κB activation requires proteasomal activity, our data suggest that NF-κB inhibition is not the mechanism by which DSF:Cu sensitises cancer cells to ionising radiation.

We report that treatment with DSF:Cu reduced CDK1 expression levels, caused a G2 arrest and the failure to enter mitosis. CDC25 phosphatase is responsible for the dephosphorylation and activation of CDK1, which leads to progression through mitosis. CDC25 phosphatase contains a cysteine residue (C473) involved in its catalytic domain [48]. Given the ability of DSF to form disulfide bonds, a possible scenario is the formation of a mixed disulfide between DSF and C473 of CDC25. The resulting sustained phosphorylated state of CDK1, in conjunction with reduced levels of CDK1, prevents progression through mitosis and promotes accumulation of cells in G2. CDK1 is also an effector of S phase checkpoint activation following treatment with DNA damaging agents such as cisplatin and ionising radiation [34]. It follows that CDK1 depletion by DSF:Cu cannot explain inhibition of DNA synthesis. Interestingly, many DNA replication enzymes contain iron-sulfur clusters or thiol residues susceptible to mixed disulfide formation with DSF [49, 50]. Furthermore, the thiol-containing small molecules glutathione, glutaredoxin and thioredoxin are required to supply electrons to ribonucleotide reductase in order to convert ribonucleotides into deoxyribonucleotides for DNA synthesis [51]. It has also been reported that non-protein thiol levels increase during S phase [52]. Therefore, by virtue of DSF’s ability to form mixed disulfides with cellular components [53], the interaction of DSF:Cu with thiol-containing molecules may explain its inhibitory action on DNA synthesis.

It has been reported that CDK1 depletion leads to sensitisation of S phase cells to DNA damaging agents such as cisplatin and ionising radiation [34]. However, our observation that DSF:Cu depleted CDK1 levels but failed to sensitise S phase cells to irradiation precludes CDK1 depletion as the mechanism for DSF:Cu-induced radiosensitisation. Instead, the lack of sensitisation of S phase cells to ionising radiation by DSF:Cu was attributed to the inhibition of DNA replication. This hypothesis is consistent with reports suggesting that the mechanism of radiation-induced cell kill in S phase is dependent on a progressing replication fork, which, upon collision with radiation-induced DNA single strand breaks (SSB), generates lethal DSB. For instance, it has been shown that the collision of the replication fork with SSB created by treatment with topotecan generates lethal DSB which synergises with ionising radiation [54]. Similarly, radiosensitisation by olaparib, an inhibitor of SSB repair, was enhanced in populations synchronised in S phase and abrogated by concomitant exposure to the DNA polymerase α inhibitor aphidicolin [36].

Gemcitabine is a known radiosensitiser through the following hypothetical mechanisms. As a prodrug, the nucleoside analogue gemcitabine requires phosphorylation by deoxycytidine kinase (dCK) for its incorporation into DNA. It has been reported that ionising radiation increases dCK expression and activity in a dose- and time-dependent manner [55]. Secondly, it has been suggested that gemcitabine is a radiosensitiser through the inhibition of ribonucleotide reductase-mediated supply of dATP for DNA repair [56] and through the accumulation of cells in early S phase, a radiosensitive phase of the cell cycle [57]. However, it has been shown that glutathione confers upon cancer cells resistance to gemcitabine [58]. Therefore, increased oxidative stress resulting from the interaction between DSF:Cu and glutathione [53] could sensitise cancer cells to gemcitabine treatment. In preclinical studies, DSF, and its derivative pyrrolidine dithiocarbamate, have been successfully used to enhance the efficacy of gemcitabine, notably through NF-κB inhibition and generation of oxidative stress [5, 37, 38]. Alternatively, DSF:Cu has been shown to inhibit ALDH activity [5, 39, 40], the formation of tumorspheres *in vitro* [5] and the growth of ALDH-positive xenografts *in vivo* [6], indicating the toxicity of DSF:Cu to cancer stem cells. We cannot speculate whether enhancement of toxicity by the combination of DSF:Cu, ionising radiation and gemcitabine is mediated by ALDH inhibition and the depletion of cancer stem cells because, in our study, we cultured our cells in conditions not optimised for cancer stem cell enrichment. We complement these studies by suggesting an alternative mechanism by which gemcitabine enhances DSF:Cu toxicity, namely the targeting, by gemcitabine, of S phase cells which are resistant to radiosensitisation by DSF:Cu.

Clonogenic cell kill achieved by the combined treatment consisting of DSF:Cu and ionising radiation was enhanced by pre-administration or subsequent administration of gemcitabine in SK-N-BE(2c), UVW, SH-SY5Y and T98G cells. However, the simultaneous treatment with gemcitabine, DSF:Cu and ionising radiation resulted in enhanced clonogenic cell kill in SK-N-BE(2c) cells, but not in UVW, SH-SY5Y and T98G cells. Gemcitabine toxicity to S phase cells depends on its incorporation into DNA and DSF:Cu inhibits DNA synthesis. This may explain the failure of gemcitabine to enhance the efficacy of the combination treatment consisting of ionising radiation and DSF:Cu when administered simultaneously. Furthermore, it has been suggested that gemcitabine exerts cytotoxic actions in S phase by virtue of binding to critical thiols of ribonucleotide reductase [19]. Since DSF:Cu also interacts with thiols [18, 53], the competition for these binding sites may hinder enhancement of treatment efficacy.

Our observation of modulation of cell cycle progression by DSF:Cu suggested that enhancement of the efficacy of DSF:Cu combined with ionising radiation may be achieved through the application of cytotoxic drugs acting preferentially on cells in S phase. Indeed, decreased clonogenic survival was obtained by the inclusion of the nucleoside analogue gemcitabine with DSF:Cu and ionising radiation treatments. However, the radiosensitising mechanism of DSF:Cu deserves further study. In particular, critical thiols in effectors of DNA repair, cell cycle checkpoint and other pathways linked to the cellular response to ionising radiation are possible targets for DSF:Cu-induced radiosensitisation. Recently, DSF:Cu treatment has been associated with decreased expression of genes involved in DNA repair [31]. The knowledge of the radiosensitising mechanism of DSF:Cu could be used to refine combination treatments and to define cancer patient sub-groups most likely to benefit from DSF:Cu and ionising radiation therapy.

It is noteworthy that the repurposing of well-established drugs such as DSF to cancer treatment has received recent interest [59]. In comparison to newly designed drugs, the advantages of DSF:Cu include cheaper and shorter clinical development due to prior knowledge of its favourable toxicological, pharmacokinetic and pharmacodynamic profiles. The clinical experience of DSF and gemcitabine treatments should facilitate the introduction of their combined use in clinical trials of radiotherapy.

## MATERIALS AND METHODS

### Cell culture

The neuroblastoma-derived cell lines SK-N-BE(2c) and SH-SY5Y and the glioma-derived cell line T98G were purchased from the American Type Culture Collection. SK-N-BE(2c) cells were maintained in Dulbecco-modified Eagle medium supplemented with 15% (v/v) foetal bovine serum (FBS, Autogen Bioclear, Wiltshire, UK), 2 mM L-glutamine and 1% (v/v) non-essential amino acids at 37°C in a 5% CO_2_ atmosphere. SH-SY5Y cells were maintained in Dulbecco-modified Eagle medium supplemented with 10% (v/v) foetal bovine serum FBS and 2 mM L-glutamine at 37°C in a 5% CO_2_ atmosphere. The glioma-derived cell lines UVW [60] and T98G were maintained in minimum essential medium supplemented with 10% (v/v) FBS and 2 mM L-glutamine at 37°C in a 5% CO_2_ atmosphere. Unless otherwise indicated, all cell culture reagents were obtained from Thermo Scientific (Paisley, UK).

### Drug treatment and irradiation procedure

Gemcitabine, DSF, copper chloride, NAC and Tiron were purchased from Sigma-Aldrich (St Louis, MO, USA). Gemcitabine and DSF were dissolved in DMSO. The cells were exposed to a maximum concentration of 0.1 % (v/v) DMSO. Copper chloride was dissolved in distilled water. NAC and Tiron were dissolved in culture medium. DSF:Cu treatment consisted of equimolar concentrations of DSF and copper in serum-free culture medium in order to control for copper concentration [10]. Cells were irradiated using an RS225 irradiator (Xstrahl, Surrey, UK) at a dose-rate of 1.64 Gy/min. For combination treatment consisting of gemcitabine, DSF:Cu and ionising radiation, three schedules of treatment administration were assessed (Supplementary Figure 2). Schedule 1 consisted of pre-treatment of cells with gemcitabine for 24 h immediately followed by treatment with DSF:Cu for 8 h. The cells were irradiated at the commencement of exposure to DSF:Cu. Schedule 2 consisted of pre-treatment of cells with DSF:Cu for 8 h, with irradiation at the commencement of exposure to DSF:Cu, immediately followed by treatment with gemcitabine for 24 h. Schedule 3 consisted of simultaneous treatment of cells with gemcitabine for 24 h and DSF:Cu for 8 h, with irradiation at the commencement of drug treatment.

### Clonogenic assay

After treatment, the medium was removed and the cells were washed twice with phosphate-buffered saline (PBS), suspended by treatment with trypsin and counted using a haemocytometer. For every treatment, 600 SK-N-BE(2c), 250 UVW, 1,000 SH-SY5Y and 700 T98G cells were seeded in triplicate in 5.3 cm diameter dishes (Thermo Scientific, Paisley, UK) and incubated at 37°C in 5% CO_2_. When the colonies comprised more than 50 cells, medium was removed and the colonies were fixed in 50% methanol (v/v) in PBS and stained with 1% (v/v) crystal violet in PBS before counting.

### Isobologram analysis

Isobologram analysis at the 50% level of effect was performed in order to determine the nature of the interaction between DSF:Cu and ionising radiation treatment according to the method of Steel and Peckham [61]. Modes IIa and IIb were chosen to define the envelope of additivity because it accounts for non-linearity of the dose-effect curves for both DSF:Cu and ionising radiation. For mode IIa, a series of ionising radiation doses that produces a 0, 10, 20, 30, 40 and 50% reduction of clonogenicity was calculated using the median effect principle, as described previously [62]. This series of effects is then used as the initial level of cytotoxicity from which the increment of the concentration of DSF:Cu that produces a total level of toxicity of 50% was calculated. For mode IIb, a series of DSF:Cu concentrations that produces a 0, 10, 20, 30, 40 and 50% reduction of clonogenicity was calculated. This series of effects is then used as the initial level of cytotoxicity from which the increment of the ionising radiation dose that produces a total level of toxicity of 50% was calculated.

The coordinates of the isoeffect points were determined using the ionising radiation survival curves obtained in the absence and in the presence of various concentrations of DSF:Cu. Firstly, the surviving fractions were plotted against the dose of ionising radiation in the absence and in the presence of 0, 0.1, 0.2 and 0.3 μM concentrations of DSF:Cu. The doses of ionising radiation producing a 50% reduction in clonogenicity (IC_50_ value) in the presence of 0, 0.1, 0.2 and 0.3 μM of DSF:Cu were calculated using the median effect principle. The pairs consisting of the IC_50_ value for ionising radiation in the presence of a selected concentration of DSF:Cu were the coordinates of the isoeffect points at the 50% level of cytotoxicity. Secondly, the analogous procedure is performed using survival curves obtained in response to DSF:Cu treatment in the presence of 0, 1, 2 and 3 Gy ionising radiation.

If the isoeffect points lay to the left of the envelope of additivity, the response was supra-additive. If the isoeffect points lay within the envelope of additivity, the response was additive. If the isoeffect points lay to the right of the envelope of additivity, the response was infra-additive.

### Combination index analysis

When experimental design did not include treatment with incremental doses of each component, the nature of the interaction between the components of a combination treatment was assessed by the calculation of the combination index. The procedure has been described previously [62]. Briefly, the median effect equation was used to calculate the doses of each component of the combination treatment corresponding to a given effect. The dose reduction index was then calculated for each agent of the combination by dividing the dose within the combination by the dose as a single agent that induced the same kill. The combination index (CI) was calculated as the sum of the dose reduction indices of each component of the combination.

### Chymotrypsin-like activity assay

Chymotrypsin-like activity of the proteasome was measured using Proteasome-Glo™ Chymotrypsin-Like, Trypsin-Like and Caspase-Like Cell-based Assay kit (Promega, US). Exponentially growing cells were treated in 96-well plates with DSF:Cu for 8 h at 37°C and 5% CO_2_. The cells were then washed with PBS and resuspended in 100 μl of PBS and exposed to the manufacturer’s buffer containing luciferase and the proteasome substrate Suc-LLVY-luciferin. The luminescent signal, which was a measure of proteasomal activity, was measured using a Lmax luminometer (Molecule Devices, Sunnyvale, CA).

### Cell cycle synchronisation

SK-N-BE(2c) cells were exposed twice to 20 mM thymidine for 24 h separated by incubation in thymidine-free culture medium for 24 h. UVW cells were exposed twice to 2 mM thymidine for 15 h separated by incubation in thymidine-free culture medium for 9 h. At the end of the synchronisation procedure, cells were reversibly arrested in early S phase.

### Cell cycle analysis

Cells were exposed to 10 μM bromodeoxyuridine (BrdU; BD Biosciences, Oxford, UK) for 90 min after which cellular monolayers were trypsinized, washed twice with PBS and fixed overnight in 70% (v/v) ethanol in distilled H_2_O at -20°C. Ethanol was removed by centrifugation and resuspension of the pellet in PBS. Cells were incubated in 2 M hydrochloric acid (HCl) for 20 minutes and then washed with PBS and then with PBS buffer containing 0.5% (v/v) Tween-20 (PBST). The cells were resuspended in PBS buffer containing 0.05% (v/v) Triton X-100 and BrdU antibody (BD Biosciences, Oxford, UK) for 20 min at room temperature. Excess antibody was removed by centrifugation and resuspension in PBST. The cells were then resuspended in 200 μl of PBS containing 20 μg/ml of propidium iodide (Sigma-Aldrich, St Louis, MO, USA) and 200 μg/ml of RNase A (Qiagen Ltd., West Sussex, UK) for at least 10 min before flow cytometry using a FACScan analyzer (BD Biosciences, Oxford, UK). Data were analyzed using the FlowJo software (BD Biosciences, Oxford, UK).

### Immunoblotting

Whole cellular protein extracts were resolved in reducing and denaturing conditions by sodium dodecyl sulphate polyacrylamide gel electrophoresis for the determination of expression levels of ATM, ATM phosphorylated at serine 1981 (ATM^1981^), CDK1 and phosphorylated CDK1 at tyrosine 15 (CDK1^15^). Proteins were transferred onto polyvinylidene fluoride Immobilon-P® membranes (Millipore, UK). Membranes were blocked with 7.5% (w/v) milk (CDK1, ATM, ATM^1981^ and β-actin) or bovine serum albumin (CDK1^15^), then incubated overnight at 4°C with primary antibodies against CDK1 (1:1,000; mouse monoclonal, Millipore, UK), CDK1^15^ (1:50,000; rabbit polyclonal, Millipore, UK), ATM (1:1,000; mouse monoclonal, Abcam, UK), ATM^1981^ (1:1,000; mouse monoclonal, Novus Biologicals, UK) and loading controls β-actin (1:50,000; mouse monoclonal, Abcam, UK). Membranes were then washed and incubated with horseradish peroxidase-conjugated secondary anti-mouse antibodies (1:2,000; Cell Signalling, UK) or anti-rabbit antibody (1:2,000; Santa-Cruz, Heidelberg, Germany) to enable detection using enhanced chemolumiscence (Thermo Scientific, UK). The densitometric analysis was carried out using ImageJ software (National Institute of Health, USA). The intensity of the band above background was recorded, normalised to that of β-actin and expressed as a fold-change in comparison with untreated controls.

### Circular dichroism

Calf thymus DNA (Sigma-Aldrich, MO, USA) was denatured at 90°C for 5 min and allowed to cool and anneal at room temperature for 40 min. PBS, ethidium bromide (EtBr; Sigma-Aldrich, MO, USA) or DSF:Cu were added to 0.05 mg/ml calf thymus-DNA in 1 ml PBS prior circular dichroism (CD) spectra acquisition using a Jasco-600 spectropolarimeter (Japan Spectroscopic Co., Tokyo, Japan). CD spectra (225 to 300 nm) were collected using a quartz cell with a path length of 0.5 cm. Alternatively, CD spectra (225 to 300 nm) were collected from 85 to 20°C at 5°C increments.

## SUPPLEMENTARY MATERIALS FIGURES


