# Supplementary material for: Cell cycle specific radiosensitisation by the disulfiram and copper complex
Source: Oncotarget. 2017 Jul 25;8(39):65900–16. doi: 10.18632/oncotarget.19539 (PMC5630381; doi:10.18632/oncotarget.19539)
Supplement: Supplementary file 1 [file oncotarget-08-65900-s001.pdf]

Cell cycle specific radiosensitisation by the disulfiram and copper complex

SUPPLEMENTARY MATERIALS

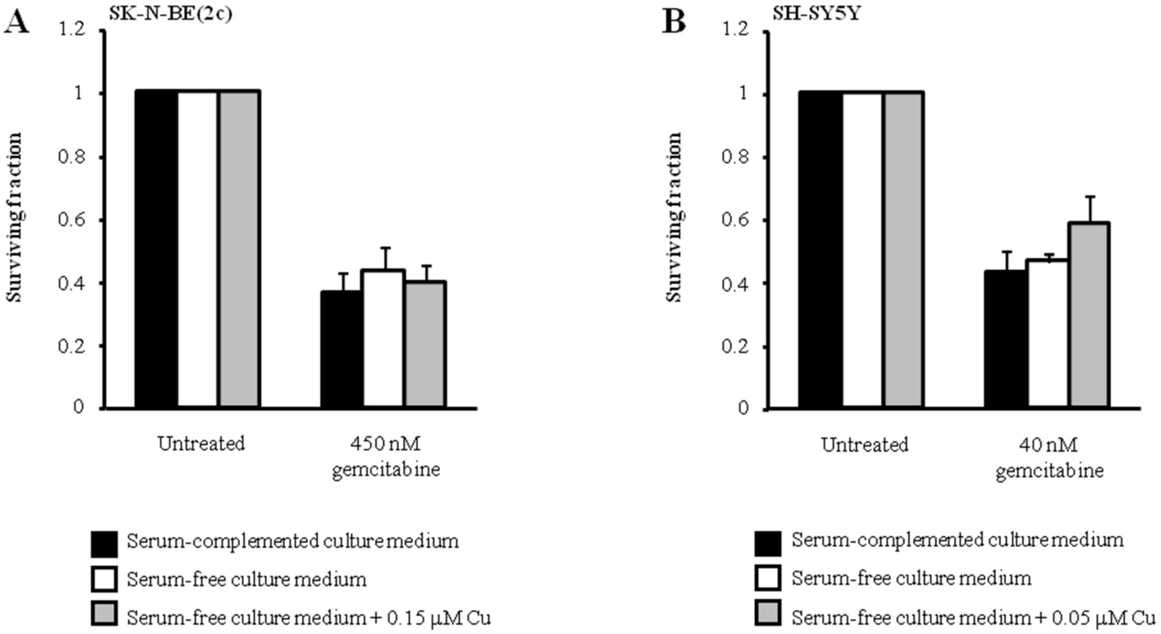

Supplementary Figure 1: The effect of Cu on the clonogenic survival of SK-N-BE(2c) and UVW cells following treatment with gemcitabine. (A) Unsynchronised SK-N-BE(2c) cells were exposed to 450 nM gemcitabine in serum-complemented culture medium, serum-free culture medium which does not contain Cu and in serum-free culture medium supplemented with 0.15 µM Cu. **(B)** Unsynchronised SH-SY5Y cells were exposed to 40 nM gemcitabine in serum-complemented culture medium, serum-free culture medium which does not contain Cu and in serum-free culture medium supplemented with 0.05 µM Cu. Data are means ± SEM, n=3.

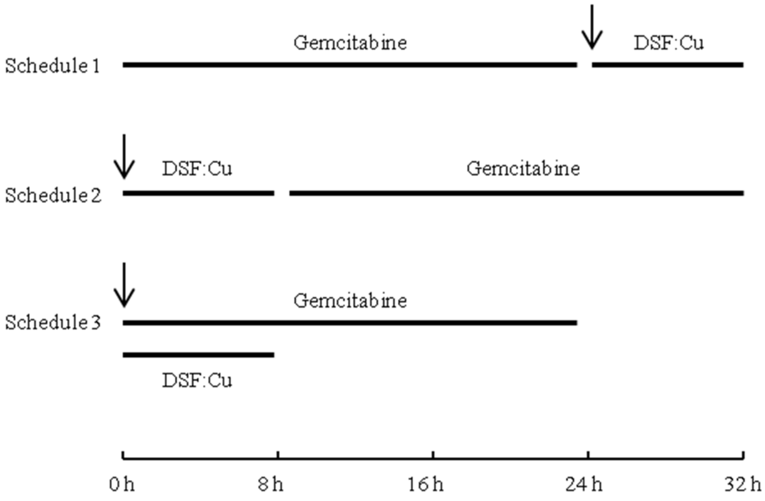

Supplementary Figure 2: The treatment schedules consisting of gemcitabine, DSF:Cu and ionising radiation. The arrows indicate the time of irradiation.
